# Metaheuristic algorithm integrated neural networks for well-test analyses of petroleum reservoirs

**DOI:** 10.1038/s41598-022-21075-w

**Published:** 2022-10-03

**Authors:** Rakesh Kumar Pandey, Shrey Aggarwal, Griesha Nath, Anil Kumar, Behzad Vaferi

**Affiliations:** 1 Department of Petroleum and Energy Studies, School of Engineering and Technology, DIT University, Dehradun, India; 2 Data Science Research Group, School of Computing, DIT University, Dehradun, India; 3 Department of Chemical Engineering, Shiraz Branch, Islamic Azad University, Shiraz, Iran; 4 Department of Advanced Calculations, Chemical, Petroleum, and Polymer Engineering Research Center, Shiraz Branch, Islamic Azad University, Shiraz, Iran

**Keywords:** Engineering, Fossil fuels

## Abstract

In recent years, well-test research has witnessed several works to automate reservoir model identification and characterization using computer-assisted models. Since the reservoir model identification is a classification problem, while its characterization is a regression-based task, their simultaneous accomplishment is always challenging. This work combines genetic algorithm optimization and artificial neural networks to identify and characterize homogeneous reservoir systems from well-testing data automatically. A total of eight prediction models, including two classifiers and six regressors, have been trained. The simulated well-test pressure derivatives with varying noise percentages comprise the training samples. The feature selection and hyperparameter tuning have been performed carefully using the genetic algorithm to enhance the prediction accuracy. The models were validated using nine simulated and one real-field test case. The optimized classifier identifies all the reservoir models with a classification accuracy higher than 79%. In addition, the statistical analysis approves that the optimized regressors accurately perform the reservoir characterization with mean relative errors of lower than 4.5%. The minimized manual interference reduces human bias, and the models have significant noise tolerance for practical applications.

## Introduction

Underground fossil resources, including (gas^1^, gas condensate^2^, oil^3^, and coal^4^) are essential to satisfy energy demand in domestic, transportation, and industrial applications. These energy resources must be managed efficiently so that the maximum fluid can be extracted^5^. Accurate information about these highly heterogeneous systems is a prerequisite for reservoir management. The core analysis^6^, well-logging^7^, seismic^8^, and well-testing^9^ are the main available techniques for gathering information about hydrocarbon reservoirs for efficient management. Since the well testing is a dynamic operation and helps estimate the average properties of hydrocarbon reservoir over the drainage area, it attracted high popularity in this regard. During the well-testing operation, the pressure response corresponding to a temporary change in flow rate is recorded over time. Well-test analysis is an inverse solution approach to investigate reservoir characteristics^10^. It can be achieved by developing analytical or intelligent models of the reservoir which produce similar output responses as the existing reservoir systems^11^.

The well-test data interpretation methods have always been of keen interest to researchers worldwide. The type-curve analysis of the pressure derivatives has been amongst the most popular interpretation techniques^12,13^. The predictive modeling techniques have evolved to be prominent solutions to the well-test analysis problems^14–16^.

The artificial neural network (ANN) model has been demonstrated for the first time for identifying reservoir models using pressure derivative data^17^. Kharrat and Razavi trained ANN using normalized pressure derivative data to recognize homogeneous and dual-porosity reservoir models^18^. Almaraghi and El-Banbi developed an MLP with a back-propagation algorithm for reservoir model identification^19^. Ahmadi et al. combined time-series shapelets and machine learning methods (probabilistic neural network, random forest, and support vector machines) to detect reservoir models from pressure transient signals^20^. The confusion matrix has been applied in this study to monitor classification accuracy of the suggested strategy. All these three research studies only focused on reservoirs’ model identification and made no effort to estimate the associated parameters.

On the other hand, some researchers have utilized the traditional machine learning method only to characterize the reservoirs’ parameters. The Horner plot data of build-up tests from conventional and dual-porosity reservoirs was input into the ANN model to predict the initial reservoir pressure, skin, and permeability^21^. Alajmi and Ertekin attempted to estimate naturally fractured reservoir parameters using ANN with coefficients of interpolating polynomials of pressure data and measured the model performance using mean relative error^22^. Adibifard et al. utilized the synthetic pressure transient signals of naturally fractured reservoirs to estimate permeability, wellbore storage coefficient, skin factor, interporosity flow coefficient, and storativity ratio^23^. Indeed, these studies are only applicable to estimate some key reservoir parameters and have no business with the model identification.

The derivative curve characteristics, including the radial flow regime and the hump, were used to distinguish between the infinite homogeneous and dual-porosity reservoirs, alongside estimating their parameters using three-layered ANN^24^. Since this method uses the characteristic shapes of derivative curves, it needs user knowledge/help to distinguish reservoir models.

The recent advances in computational models and deep neural networks have provided promising results in different research fields^25–30^, including well-testing analysis^31–35^. Convolutional neural networks have many parameters due to vectorizing image input, which results in increasing the computational cost and training time. Standard recurrent neural networks are unsuitable for establishing long-term dependencies across the sequence datasets. The simple architecture of ANN makes it an attractive tool for well-test analysis. Further, gradient descent^36–38^ and evolutionary^39–42^ optimization algorithms aid in improving the efficiency of the model.

However, the well-testing research has not witnessed the implementation of evolutionary optimization as genetic algorithm (GA) integrated deep structured prediction models for automatic analysis of the noisy pressure transient test data of petroleum reservoirs so far in the published research outcomes. In addition, the simultaneous performing reservoir model identification and characterization are so problematic that little research has covered that. This paper investigates GA-optimized ANN (GA-ANN) prediction models to classify homogeneous petroleum reservoirs and simultaneously estimate the associated reservoir parameters. The homogeneous reservoir models with infinite acting (HO-IA), no flow (HO-NF), and pressure supported (HO-PS) boundary conditions have been considered, and 50 × 10^3^ pressure derivative (∆p′) data for each state was obtained. Cumulatively 150 × 10^3^ labeled pressure derivative signals and their corresponding reservoir parameter, Ln (C_D_e^2S^), have been used. The classifiers and regressors comprising the fully connected layers have been developed to perform the predictions. Further, the binary coded GA has been implemented to tune the hyperparameters and select the best features from the input dataset. Later, the GA-ANN models were implemented to perform predictions using the test case data. Comparative performance validation of ANN and GA-ANN models using ten well-test data shows the superiority of GA-ANN models in reservoir classification and characterization.

## Methods

In this section, the data collection and model framework of the models have been discussed.

### Data collection

The pressure signals for HO-IA, HO-NF, and HO-PS reservoirs have been simulated using the mathematical models available in the literature^34,43^. Stehfest’s algorithm has been considered for numerical inversion of the Laplace transformation^44^. The Gaussian noise of 0–2% was added during the data simulation. Table 1 represents the details of the noise present in the data samples used for regression model training, and the ranges of characteristic parameters are available in Table 2. The time (t), pressure change (∆p), and their corresponding parameter values Ln (C_D_e^2S^) were simulated for each reservoir outer boundary condition using the python programming coded algorithm. Each test has 100 pressure data points related to the 100 timesteps.

**Table 1:** Details of noise and number of samples used for classifiers and regressors.

Reservoir type	Noise (× 10^3^)			Labels for classifiers	Total number of samples for regressors (× 10^3^)	Total number of samples for classifiers (× 10^3^)
	0%	1%	2%			
HO-IA	20	20	10	0	50	150
HO-NF	20	20	10	1	50	
HO-PS	20	20	10	2	50

**Table 2:** Range of characteristics considered for data simulation.

Range	C_D_	S	r_D_
Minimum	6.60 × 10^2^	− 1	2271
Maximum	2.70 × 10^5^	40	3211

The ∆p′ of the simulated data samples have been obtained using:

1 $$\Delta \mathrm{p{^{\prime}}} = \frac{d\left(\Delta p\right)}{d\left(\mathrm{ln}(t\right))}=t\times \frac{d\left(\Delta p\right)}{d\left(t\right)}$$

The gathered ∆p' data have been used for training the classifier and regressors. The classifiers were trained with 150 × 10^3^ labeled samples, as indicated in Table 1. Each regressor has been trained with 50 × 10^3^ data having corresponding Ln (C_D_e^2S^) values. The synthetic data set has been partitioned into 80% train, 10% validation, and 10% test set for classifiers and regressors. The data used during GA optimization of the ANN models are presented in Table 3. The data was split into 90% training and 10% validation during GA optimization.

**Table 3:** Details of noise and number of samples for GA optimization of the models.

Reservoir type	Noise (× 10^3^)			Labels for classifiers	Total number of samples for regressors (× 10^3^)	Total number of samples for classifiers (× 10^3^)
	0%	1%	2%			
HO-IA	5	5	5	0	15	45
HO-NF	5	5	5	1	15	
HO-PS	5	5	5	2	15	

**Figure 1:**
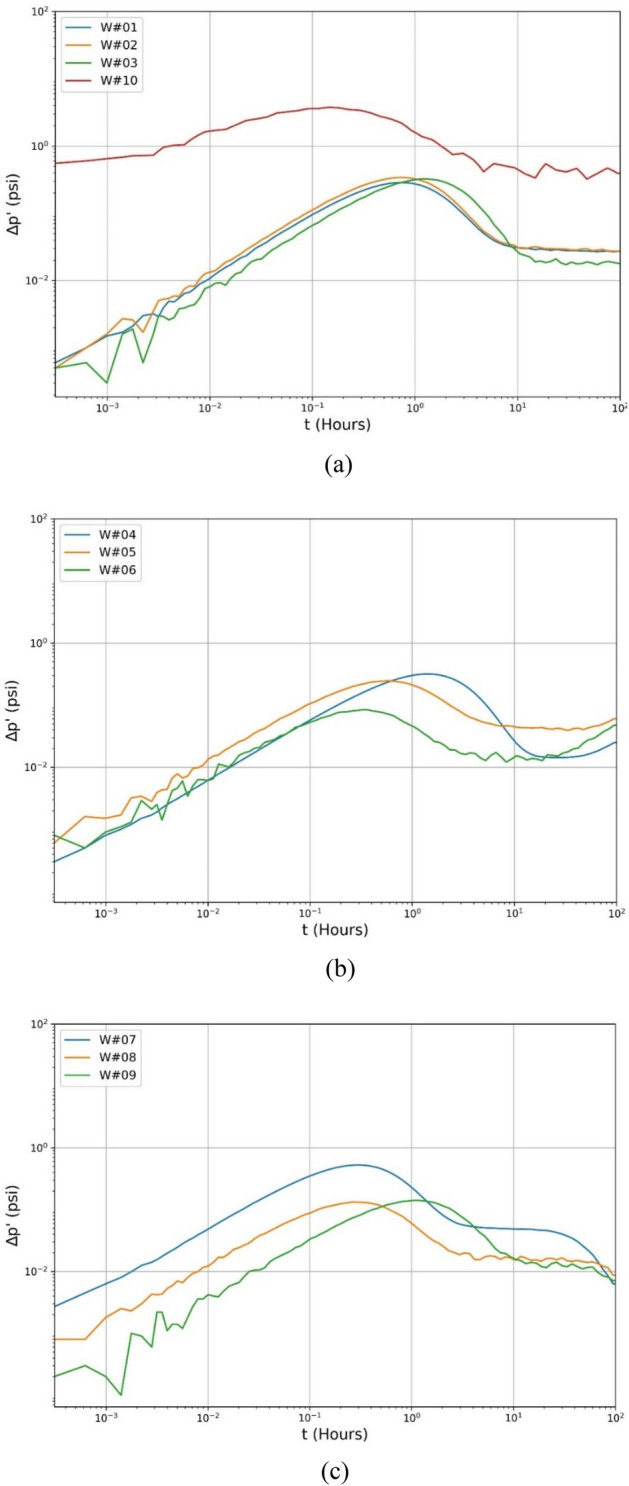
Illustration of ∆p' curves for the (**a**) HO-IA, (**b**) HO-NF, and (**c**) HO-PS reservoirs.

The optimized GA-ANN models have been trained with the data described in Table 1. The 10 test cases, including nine simulated and one real-field data^45^, have been considered to evaluate each ANN and GA-ANN model. The logarithmic plots of these cases are reproduced in Fig. 1a–c. W#01, W#04, and W#07 are smooth data, W#02, W#05, and W#08 have 1% noise, and W#03, W#06, and W#09 have 2% noise. The Gaussian noise percentage in real-field data from W#10 is unknown.

### Model framework

#### Artificial neural network

**Figure 2:**
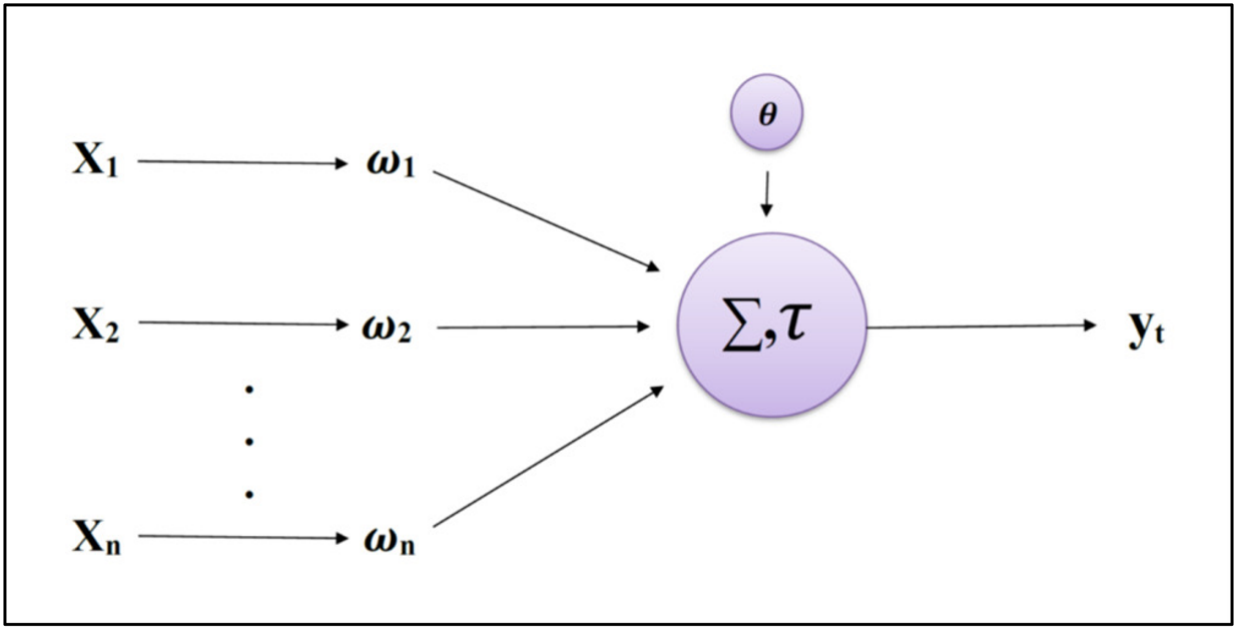
Illustration of the perceptron.

ANN are biologically inspired computer programs that learn by discovering relationships and patterns in data. The ANN architecture comprises individual units and neurons connected to weights forming sensory structures arranged in layers. The intermediate unit connections are improved during training until the prediction error is minimized. As the complexity of the model grows, the layer depth also increases, which is why it is known as the multi-layer perceptron. The purest form of ANN has one input, one hidden, and one output layer. The input layer takes the signals and transmits them to the hidden layers. The hidden layer processes the input as per the activation function, and finally, the output layer provides the prediction. Figure 2 illustrates the perceptron. The computation performed at each neuron in the layers of ANN is:

2 $${y}_{t}=\tau \left({\sum }_{i=1}^{N}{\omega }_{ti}{X}_{i}+{\theta }_{t}\right)$$

where *ω*_*ti*_ is the ith synapse of the tth neuron, *θ*_*t*_ is the bias of the tth neuron, *X*_*i*_ is the ith input of the tth neuron, *N* is the number of inputs, *τ* is the activation function, and *y*_*t*_ is the output.

**Figure 3:**
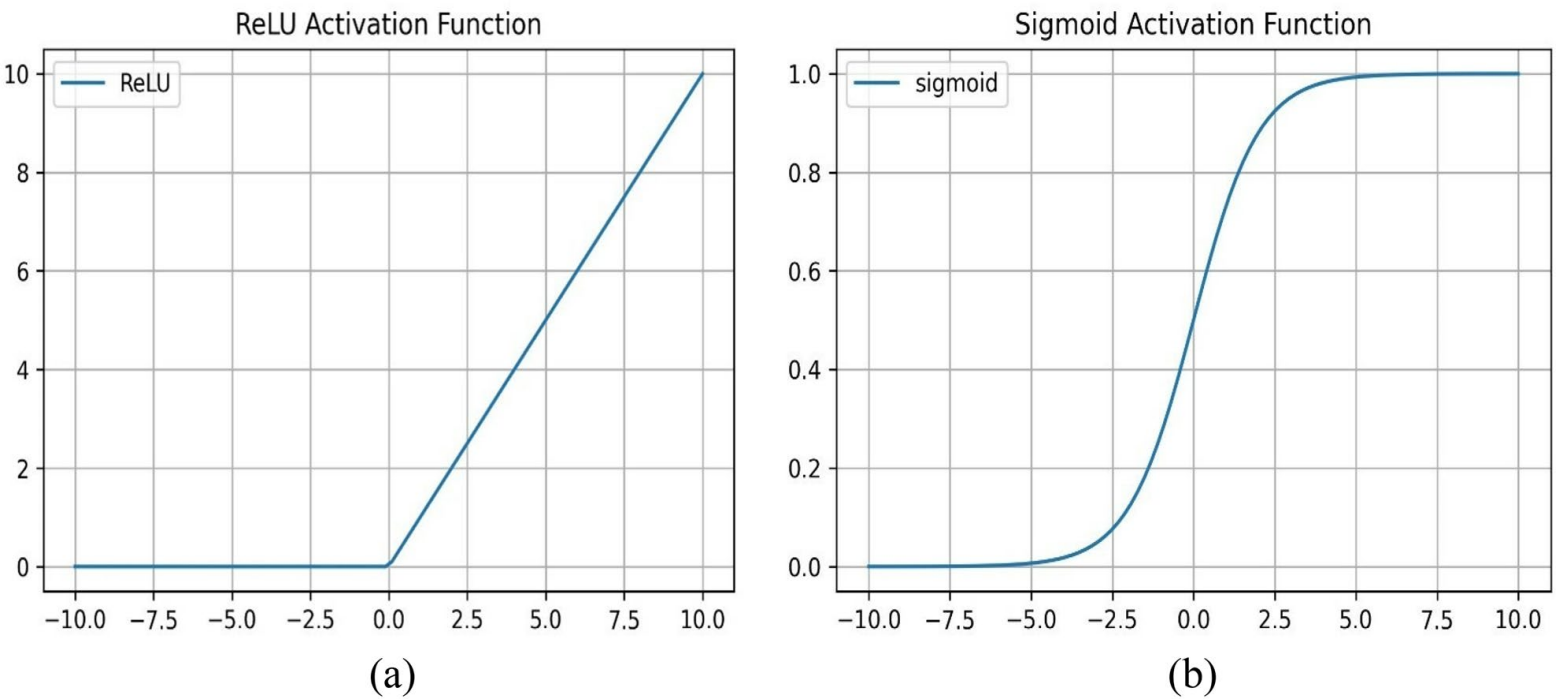
Schematic representation of (**a**) ReLU and (**b**) σ activations.

Four ANN structures (a classifier and three regressors) were first trained for 100 iterations. The six-layered classifier model has rectified linear unit (ReLU) activated hidden layers, and the output layer has sigmoid activation (σ). The three-layered regressor with ReLU activation function in a hidden layer and a linear activation function in an output layer has been trained to estimate Ln (C_D_e^2S^) associated with HO-IA, HO-NF, and HO-PS reservoirs. The activation functions decide to activate or deactivate neurons to get the desired output. The ReLu and σ activation functions convert their inputs (Z) into [0, Z] and [0, 1], as displayed schematically in Fig. 3a and b.

#### Genetic algorithm

**Figure 4:**
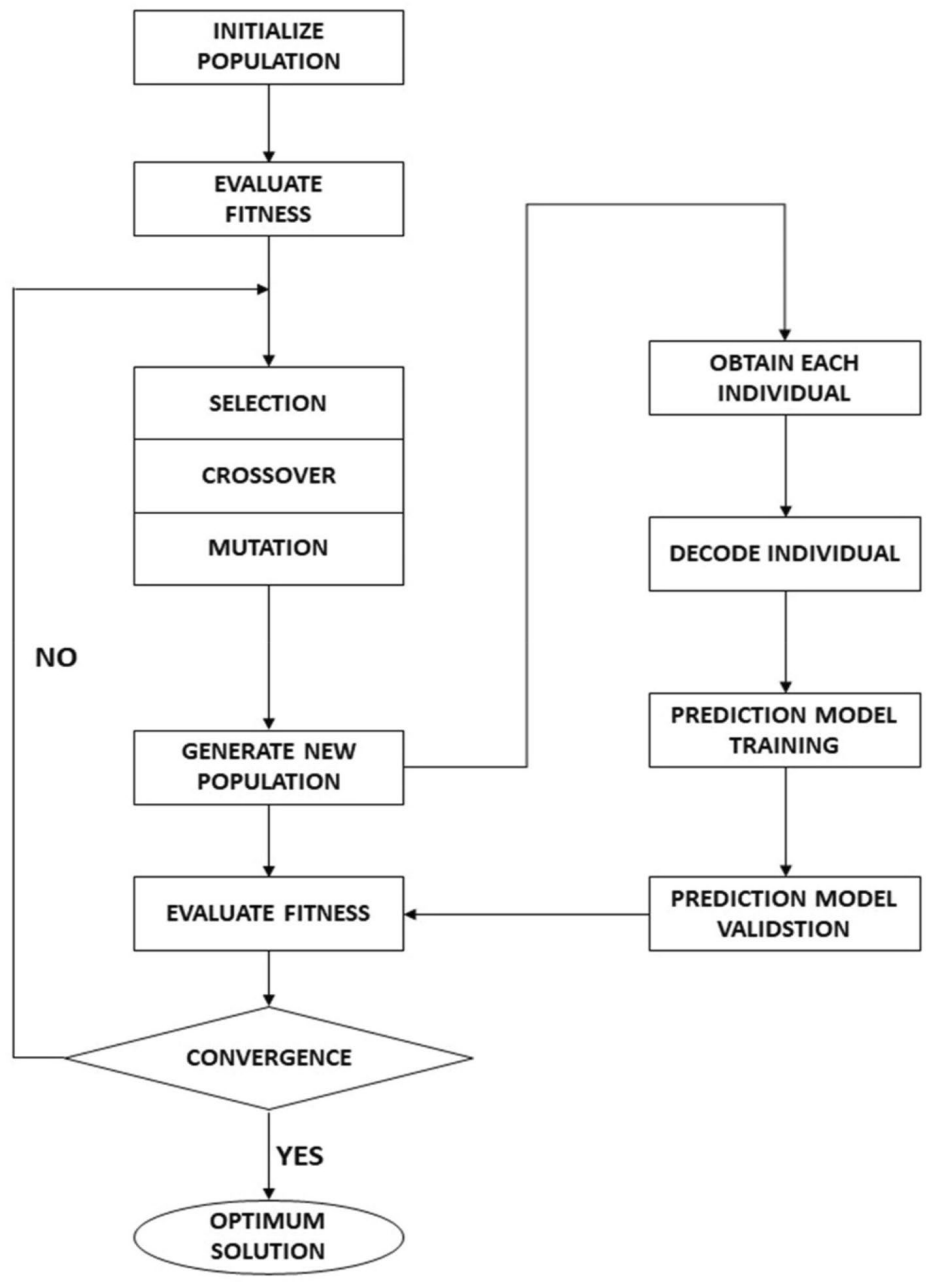
Typical procedure performed by GA.

GA is a meta-heuristic structured evolutionary optimization algorithm^46^ that combines survival of the fittest and a simplified version of the genetic inheritance process to optimize the network weights. Figure 4 represents the typical procedure performed by GA.

GA performs random sampling to generate the individual chromosomes of the initial population. The fitness function evaluates the estimation performance of the individuals using machine learning algorithms. The population evolves using genetic operators:

- *Selection* The best chromosomes are extracted from the population to generate the mating pool. Available methods to select the chromosomes include roulette wheel selection, rank-based fitness assignment, tournament selection, and elitism. This model uses tournament selection to choose the parent chromosomes for the next operator.
- *Crossover* The chromosomes of the parent individuals are swapped to generate offspring using the one-point crossover. The crossover operator ensures the inheritance of better genetic traits the future generations.
- *Mutation* These operators randomly alter the genes of the selected individual with a probability equaling the mutation rate. It preserves genetic diversity and encourages GA propagation toward global extremes.

The binary-coded GA optimization has been implemented for feature selection and hyperparameter tuning. The number of neurons in the first three hidden layers of the classifier and regressors has been tuned using the GA algorithm. Genetic operators, including tournament selection, one-point crossover, and mutation, have been used. The crossover and mutation probabilities of 0.6 and 0.2 have been considered. The iterations for 500 generations have been performed for each ANN classifier and regressor model. The GA was allowed to converge to produce the best individuals representing the optimal solution with the best fitness value. The selected features and hyperparameters from GA optimization have been used to train the optimized GA-ANN models. A total of two classifiers and six regressors have been developed, as indicated in Table 4.

**Table 4:** Details of trained classifier and regressor models.

GA optimization	Reservoir type	Regressor	Classifier
No	HO-IA	R_01_	C_01_
	HO-NF	R_02_	
	HO-CP	R_03_	
Yes	HO-IA	R_04_	C_02_
	HO-NF	R_05_	
	HO-CP	R_06_	

## Results and discussion

This section covers ANN and GA-ANN model training results and the outcomes of feature selection and hyperparameter tuning using GA. The training models' validation results have been discussed using the test cases, and comparative performance analyses were done.

### Model training performance

#### ANN models without optimization

**Figure 5:**
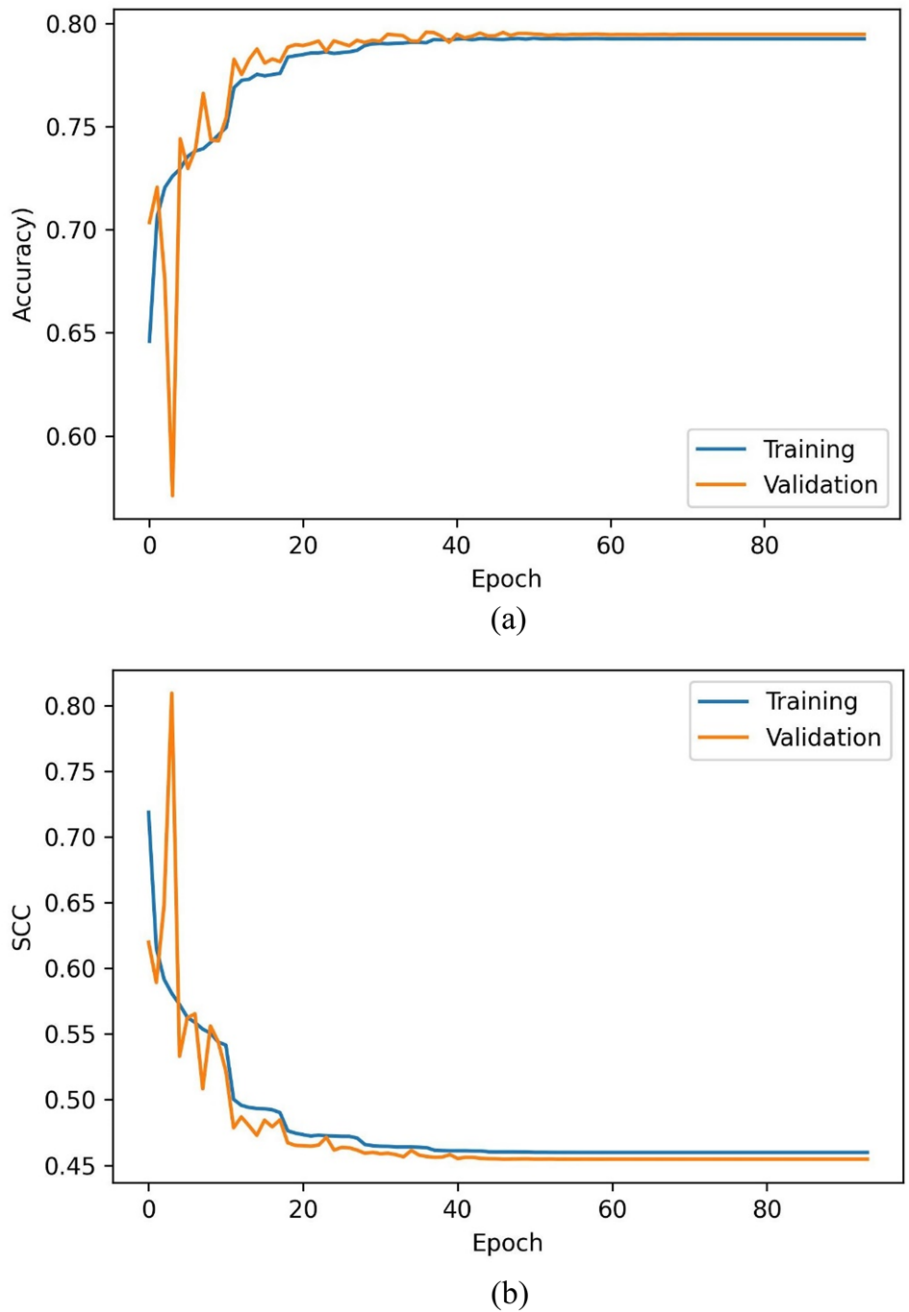
Training (**a**) accuracy and (**b**) SCC of ANN classifier.

**Figure 6:**
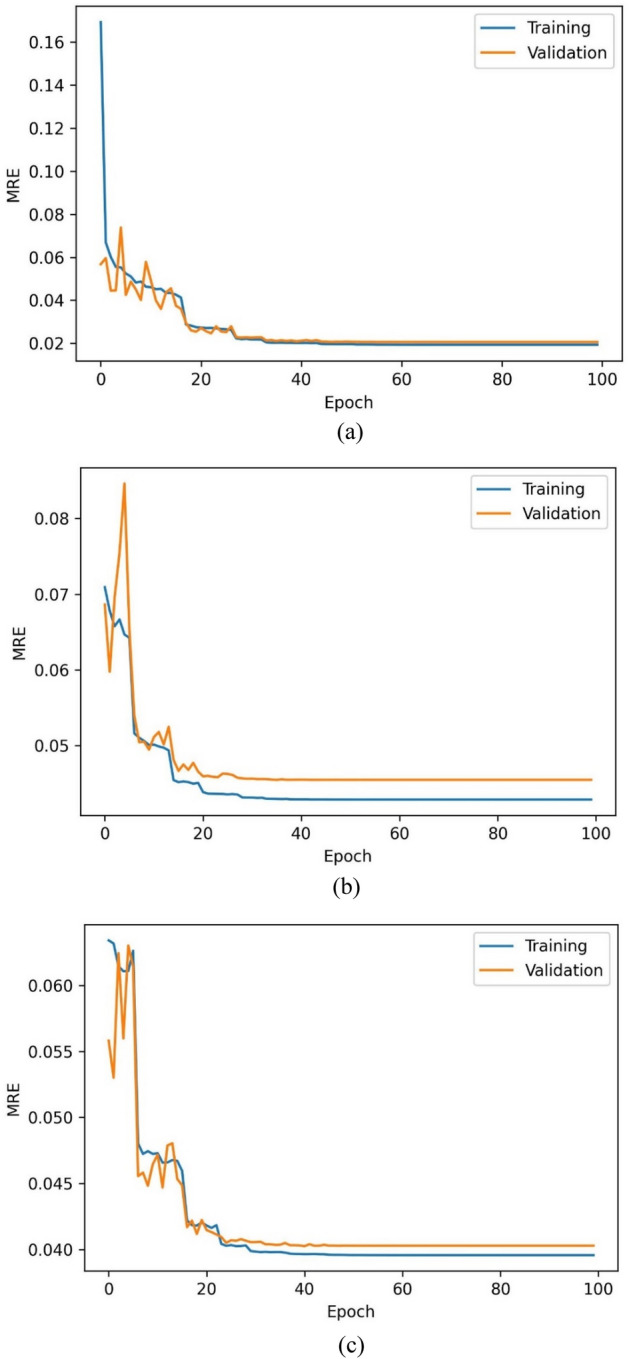
MRE of ANN regressor models for (**a**) HO-IA, (**b**) HO-NF, and (**c**) HO-PS.

The training accuracy of the C_01_ model over the train and validation set has been represented in Fig. 5a and b. The training, validation, and testing sets have achieved classification accuracies of 79.23%, 79.47%, and 79.19%. The mean relative errors (MRE) have been observed while training the regressor models [Eq. (3)]. Figure 6a–c indicate the train and validation MREs for HO-IA, HO-NF, and HO-PS reservoirs. In Fig. 5, the SCC reduced gradually, accuracy increased for forty iterations, and later became constant with slight differences between the training and validation scores. Figure 6 represents that the MRE attained low values for both training and validation. The statistics suggest that the models have been trained well without any underfitting or overfitting data.

3 $$MRE=\frac{100}{N}\sum_{i=1}^{N}\left|\frac{{y}_{i}-{\widehat{y}}_{i}}{{y}_{i}}\right|$$

where N is the total number of Data Samples; ${y}_{i}$ and ${\widehat{y}}_{i}$ are the ith target and predicted values of the model, respectively.

#### Genetic algorithm optimization of ANN models

**Figure 7:**
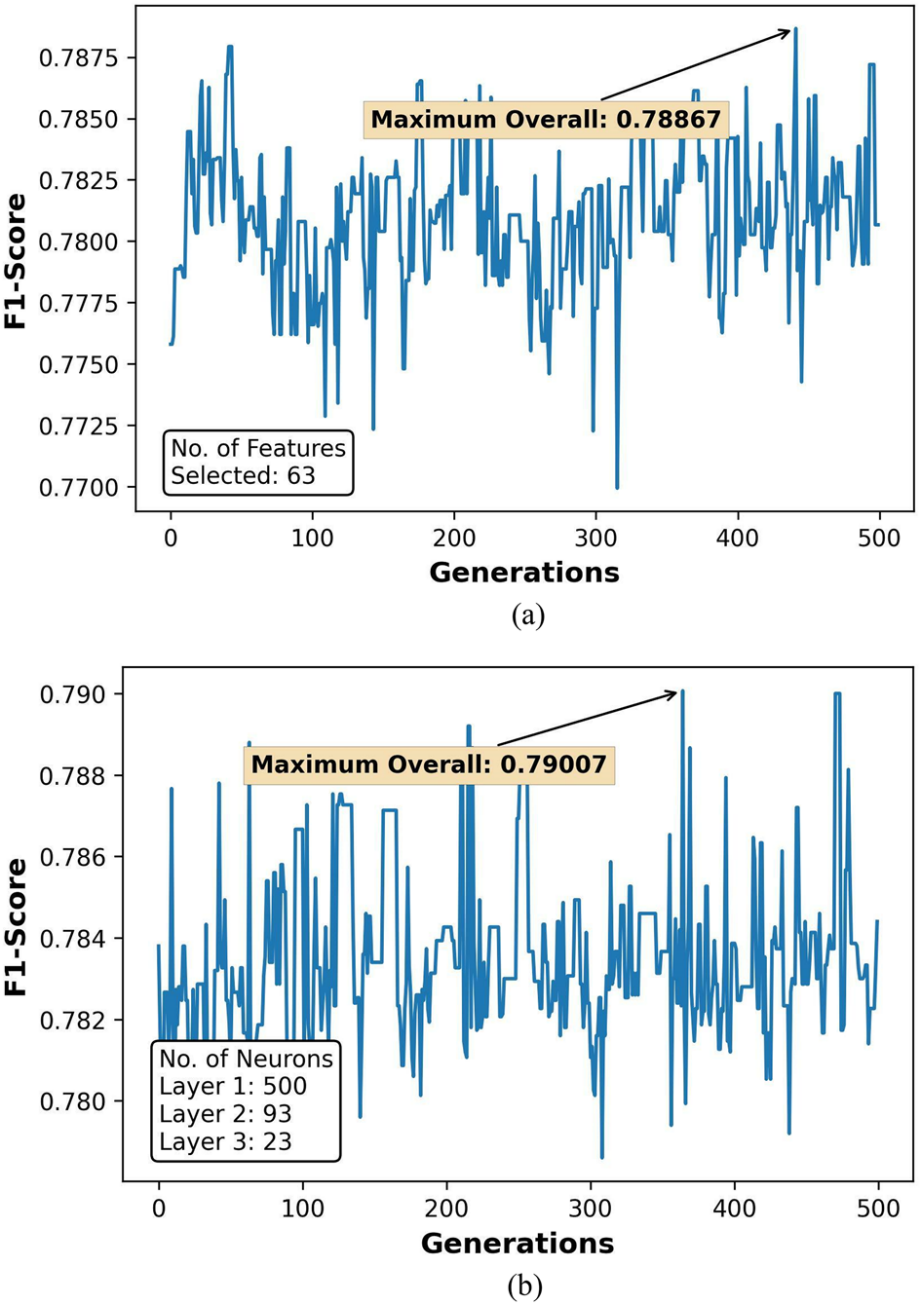
F1-Score for classifier ANN model during (**a**) feature selection and (**b**) hyperparameter tuning.

**Figure 8:**
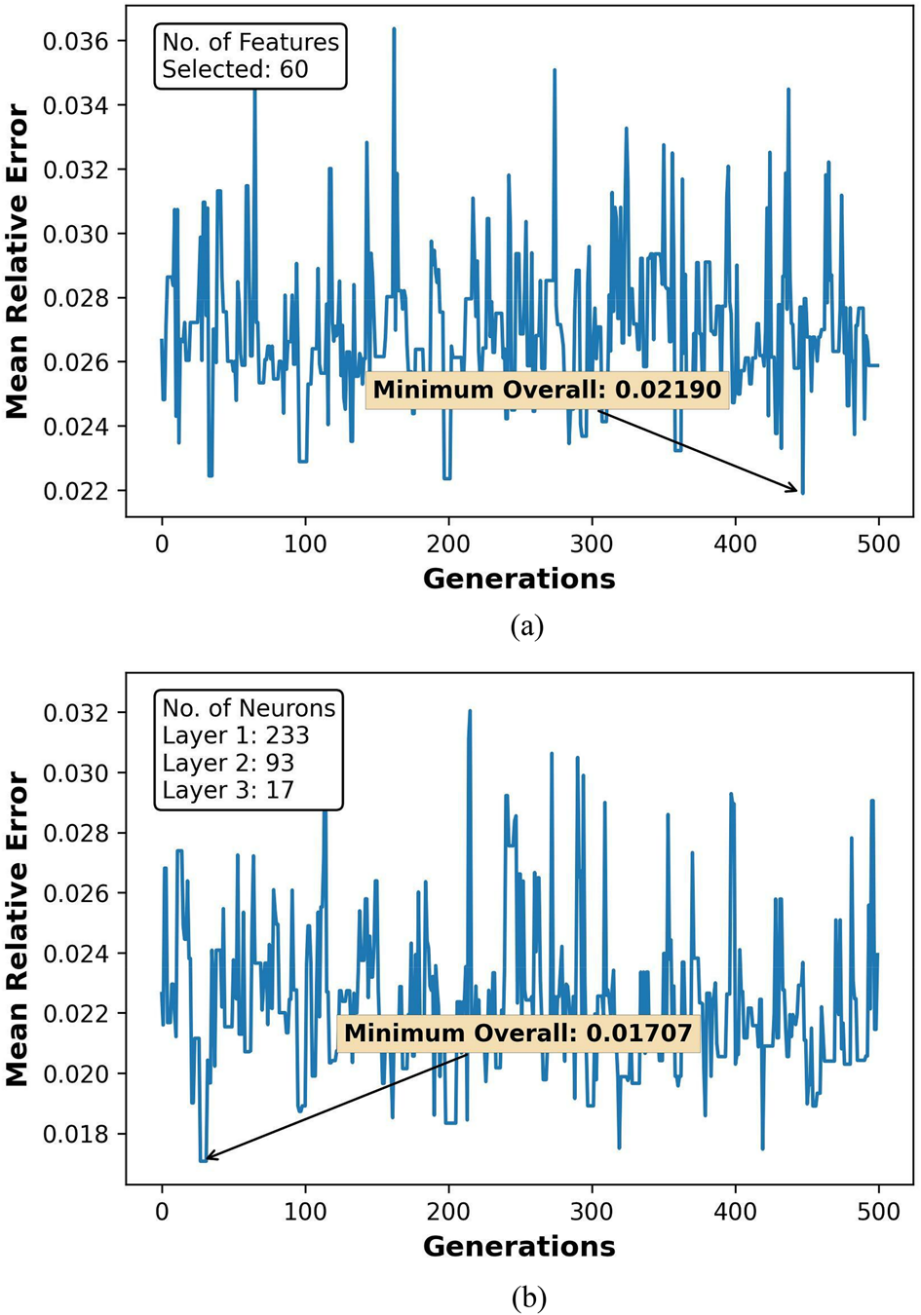
MRE for regressor ANN model during (**a**) feature selection and (**b**) hyperparameter tuning for HO-IA reservoir.

**Figure 9:**
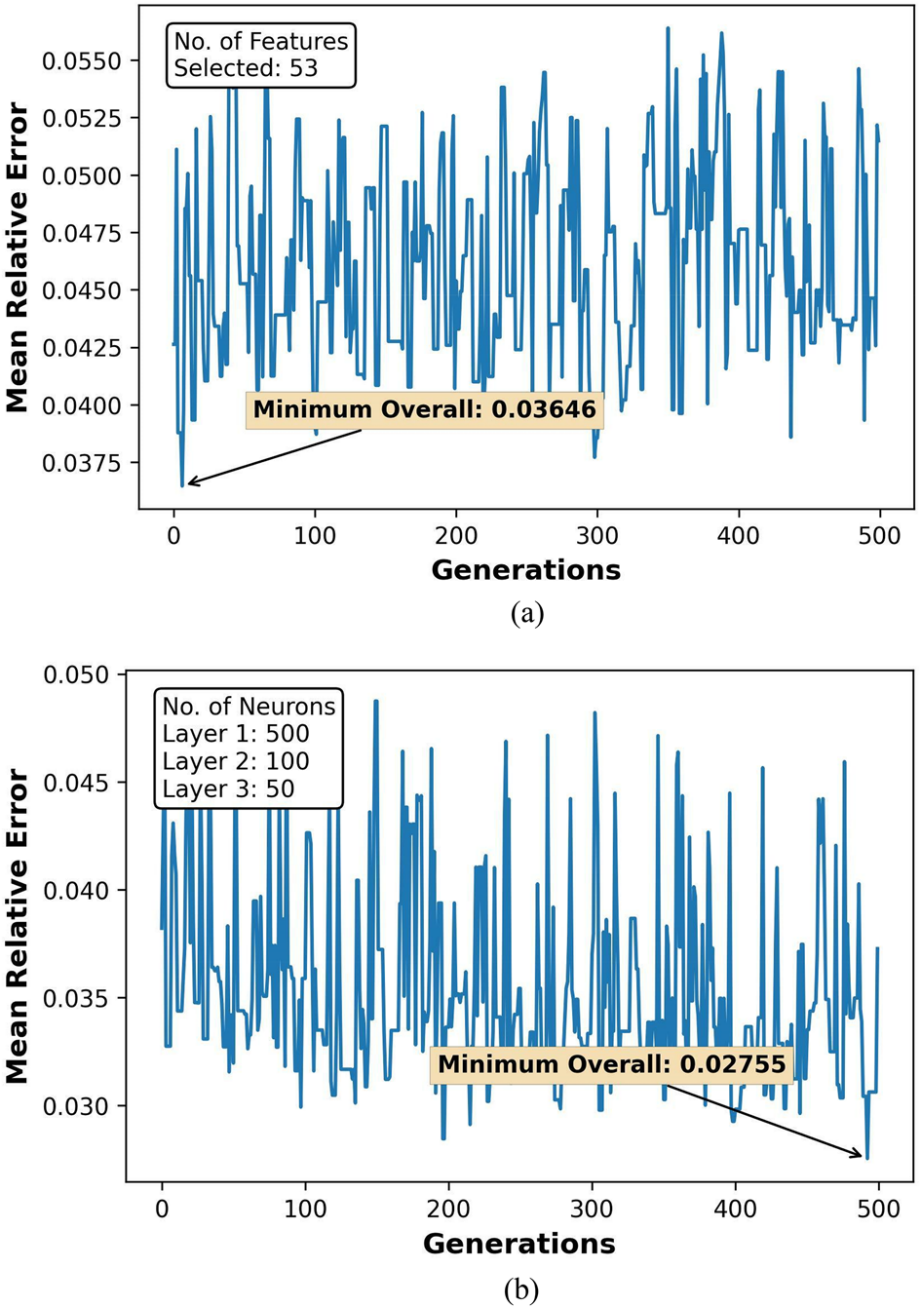
MRE for regressor ANN model during (**a**) feature selection and (**b**) hyperparameter tuning for HO-NF reservoir.

**Figure 10:**
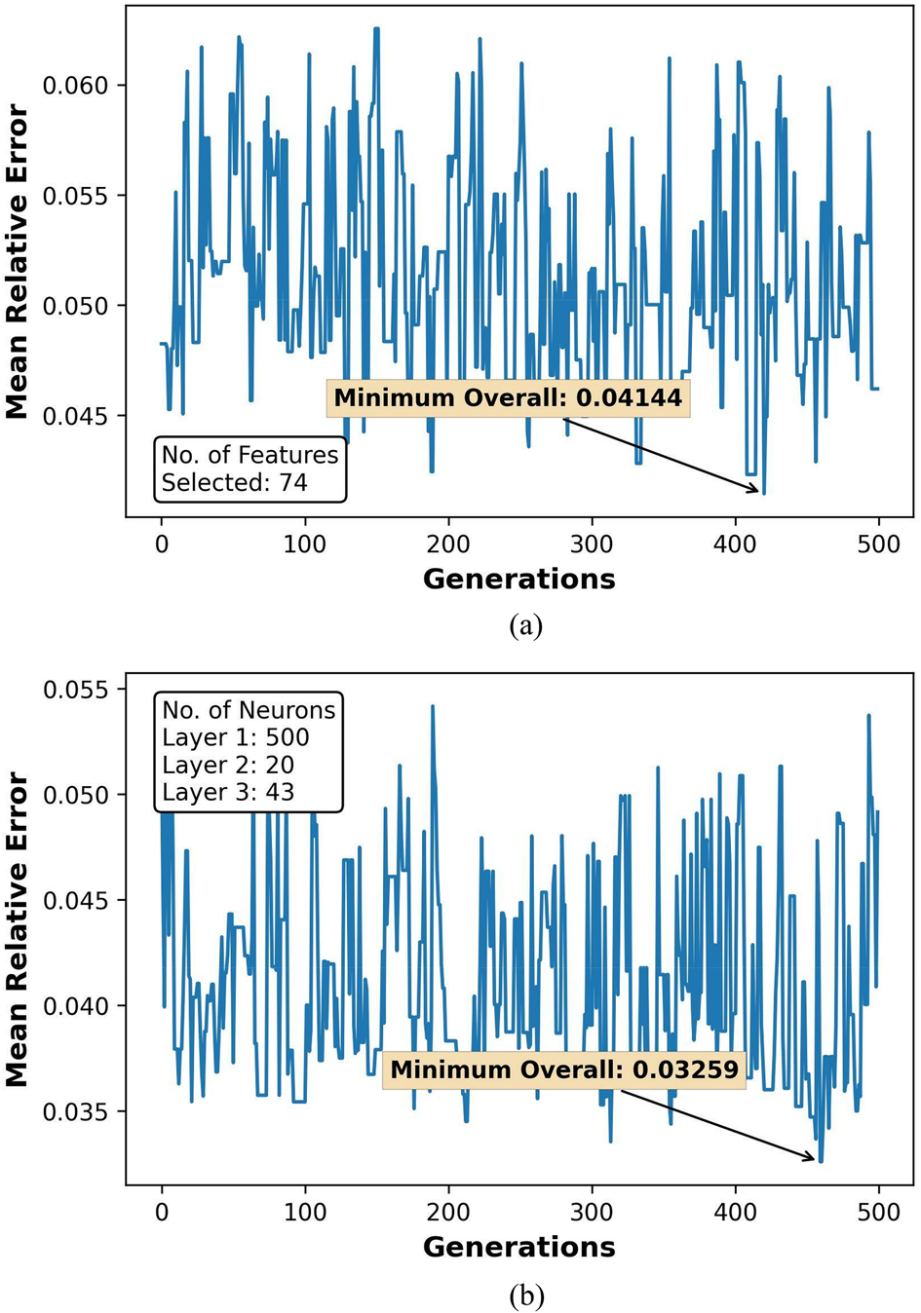
MRE for regressor ANN model during (**a**) feature selection and (**b**) hyperparameter tuning for HO-CP reservoir.

The GA optimization results for feature selection and hyperparameter tuning over 500 generations are represented in Figs. 7a,b, 8a,b, 9a,b, and 10a,b. Figures 8, 9 and 10 also show the count of selected features and the optimal neuron counts for the ANNs of each model, along with the best metric scores.

#### Optimized GA-ANN models

**Figure 11:**
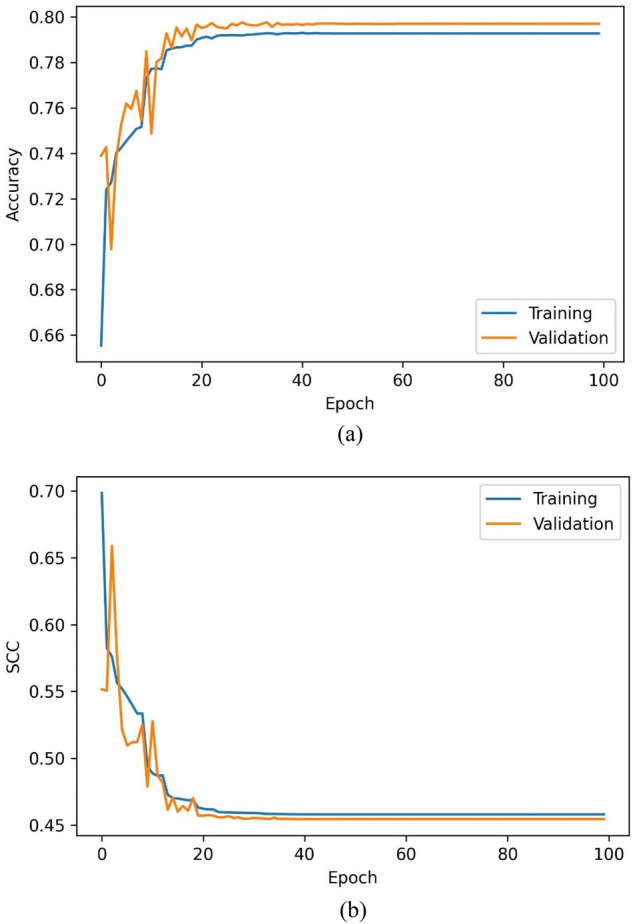
Training (**a**) accuracy and (**b**) SCC of the C_02_ model.

**Figure 12:**
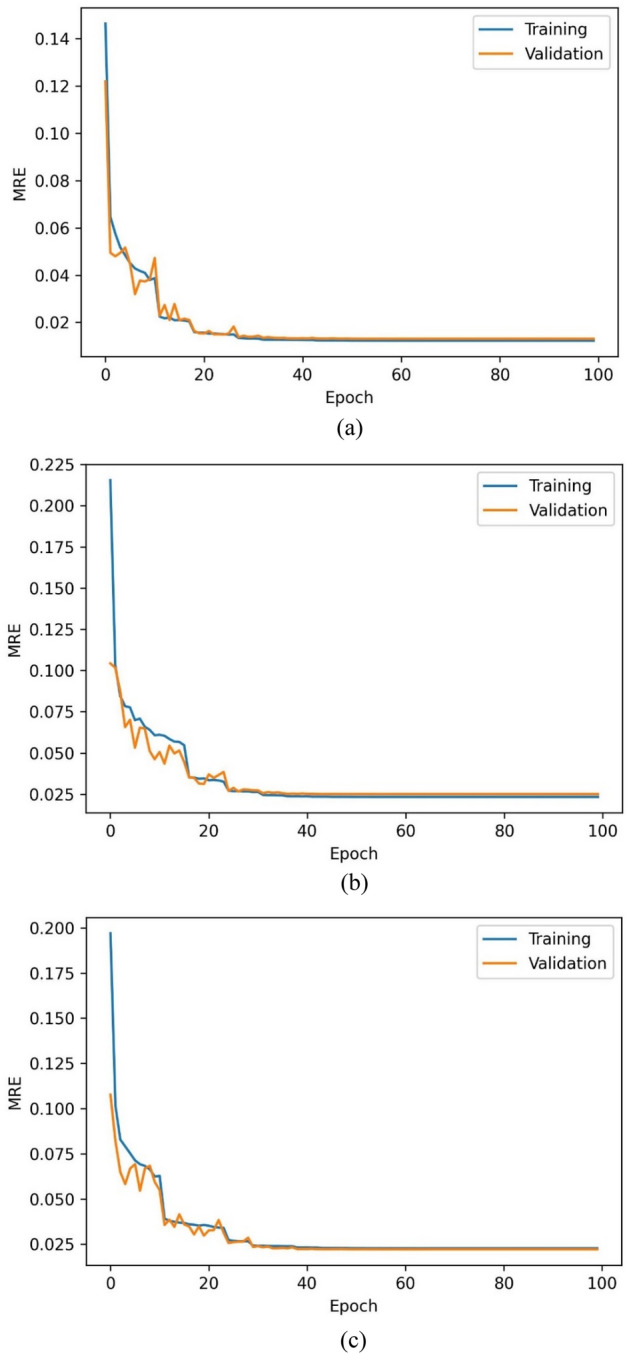
Training MRE of (**a**) R_04_, (**b**) R_05,_ and (**c**) R_06_ models.

The GA-ANN classifiers and regressors have been trained with selected features and optimized hyperparameters. Figures 11a,b and 12a–c represent the training performance of the GA-ANN classifiers and regressors over 100 iterations. Tables 5 and 6 introduce the metric scores obtained by the trained classifiers and regressors. The C_02_ achieved relatively higher accuracy than C_01_. The MREs for all the GA-ANN models have decreased compared to the ANN models showcasing improved regression performance.

**Table 5:** Accuracy and SCC values attained for the C_01_ and C_02_ model.

Model	Accuracy (%)			SCC		
	Training	Validation	Testing	Training	Validation	Testing
C_01_	79.23	79.47	79.19	0.4598	0.4546	0.4571
C_02_	79.48	79.66	79.38	0.4570	0.4545	0.4562

**Table 6:** MRE (%) attained for the regression models.

Model	MRE		
	Training	Validation	Testing
R_01_	1.93	2.05	2.09
R_04_	1.26	1.33	1.47
R_02_	4.29	4.54	4.47
R_05_	2.32	2.48	2.48
R_03_	3.95	4.02	4.07
R_06_	2.24	2.22	2.30

### Performance validation of optimized models

The test cases discussed in Section “Data collection” have been used to assess the performance of the trained models. The trained GA-ANN models predicted the reservoir models correctly for all the test cases indicating excellent classification performance. The test case data were input into the regressors for estimating their associated characteristic parameter, Ln (C_D_e^2S^). The predictions using ANN and GA-ANN models have been represented in Table 7. We observe that forecasts of seven out of ten test cases resulted in reduced error using the GA-ANN.

**Table 7:** Target and predicted values of the test cases using ANN and GA-ANN regressors.

Test case	Target	Prediction	
		ANN	GA-ANN
W#01	11.42	11.37	11.38
W#02	13.13	13.23	13.27
W#03	20.75	20.74	20.30
W#04	26.94	28.02	27.09
W#05	06.18	06.21	06.13
W#06	06.34	06.45	06.34
W#07	12.32	12.41	12.28
W#08	09.02	08.96	09.08
W#09	13.76	12.60	12.99
W#10	08.60	11.30	09.99

## Conclusions

This paper investigated the predictors' feature selection and hyperparameter tuning using GA optimization for pressure transient analysis of homogeneous reservoirs. The training results indicate that the optimized GA-ANN models yielded lower errors than the manually tuned ANN models. The optimized classification model achieved over 79% training accuracy in classifying the HO-IA, HO-NF, and HO-PS reservoirs. The GA optimization improved the regression models’ performance, and the optimized regressors predicted the characteristic parameter, Ln(C_D_e^2S^), with minimized errors. The performance of the predictive models has been validated using ten test cases. The optimized classification model accurately identified the reservoir models for all the test cases, and the regression models estimated the associated reservoir characteristics with minimized prediction errors.

Additionally, the models have adequate noise tolerance and provide computational efficiency. The proposed predictor is a robust tool for identifying and characterizing the homogeneous reservoirs from noisy real-field data with minimized human intervention. However, there is scope for improving the accuracy of the classifier model. We intend to investigate the performance of the deep structured models for the well-test analysis of heterogeneous reservoirs such as dual-porosity and composite reservoirs. Additionally, the performance comparison with other evolutionary algorithms, such as particle swarm optimization and Firefly algorithm, shall be conducted in future work.

## Data Availability

The datasets used or analyzed during the current study are available from the corresponding author upon reasonable request.
